# Accelerated Recovery Program for Patients with Polysegmental Degenerative Lumbar Spine Disease

**DOI:** 10.17691/stm2021.13.2.09

**Published:** 2021-01-01

**Authors:** A.A. Kalinin, V.Yu. Goloborodko, V.V. Shepelev, Yu.Ya. Pestryakov, M.Yu. Biryuchkov, E.E. Satardinova, V.A. Byvaltsev

**Affiliations:** Associate Professor, Department of Neurosurgery and Innovative Medicine, Irkutsk State Medical University, 1 Krasnogo Vosstaniya St., Irkutsk, 664003, Russia; Neurosurgeon, Neurosurgery Center, Road Clinical Hospital, 10 Botkin St., Irkutsk, 664005, Russia; Head of the Department of Anesthesiology and Resuscitation No.12; Doctoral Student, Department of Neurosurgery and Innovative Medicine, Irkutsk State Medical University, 1 Krasnogo Vosstaniya St., Irkutsk, 664003, Russia; Doctoral Student, Department of Neurosurgery and Innovative Medicine, Irkutsk State Medical University, 1 Krasnogo Vosstaniya St., Irkutsk, 664003, Russia; Professor, Head of the Department of Neurosurgery with Traumatology Course, West Kazakhstan Marat Ospanov Medical University, 68 Maresyev St., Aktobe, 030019, Kazakhstan; Associate Professor, Department of Reflexotherapy and Cosmetology, Irkutsk State Medical Academy for Postgraduate Education, 100 Yubileyny Microdistrict, Irkutsk, 664049, Russia; Professor, Head of the Department of Neurosurgery and Innovative Medicine, Irkutsk State Medical University, 1 Krasnogo Vosstaniya St., Irkutsk, 664003, Russia; Chief of the Neurosurgery Center, Road Clinical Hospital, 10 Botkin St., Irkutsk, 664005, Russia; Professor, Department of Traumatology, Orthopedics, and Neurosurgery, Irkutsk State Medical Academy for Postgraduate Education, 100 Yubileyny Microdistrict, Irkutsk, 664049, Russia

**Keywords:** multilevel degenerative diseases, lumbar spine, minimally invasive spinal surgery, accelerated recovery after surgery, fast-track surgery, ERAS

## Abstract

**The aim of the study** was to evaluate the effectiveness of the accelerated recovery program for patients with polysegmental degenerative diseases of the lumbar spine.

**Materials and Methods:**

This prospective study included 53 patients who underwent two-level transforaminal interbody fusion in the L_II_–S_I_ segments. Two groups were identified: in group 1 (n=24), operations were performed using the accelerated recovery program; in group 2 (n=29), open rigid stabilization was used under traditional intravenous anesthesia. The end-point parameters were the number of bed-days spent in the hospital after the operation, the number of perioperative surgical complications and adverse effects of anesthesia, and the number of re-hospitalizations within 90 days. We also recorded the time of patient activation, the level of pain around the operated zone (using a visual analogue scale), and the quality of life in the long-term postoperative period (median 18 months); the latter was assessed using the SF-36 questionnaire (physical and psychological components of health).

**Results:**

Patients under the accelerated recovery program were found to have a shorter duration of surgery and anesthesia, less blood loss, lower amounts of injected opioids, faster verticalization, and a reduced period of inpatient treatment (p<0.05 for all parameters). As compared to group 2, patients in group 1 had a lower level of pain in the surgery zone (p<0.05), better long-term indicators of the physical and psychological components of health (p<0.05), a lower number of anesthesia-associated adverse events (p<0.05), and a lower rate of postoperative complications (p<0.05). During the 90-day postoperative observation, four patients of group 2 (13.8%) were urgently referred to a medical institution for additional medical care.

**Conclusion:**

The accelerated recovery program for two-level interbody fusion showed its safety and high clinical efficiency in the treatment of patients with polysegmental degenerative diseases of the lumbar spine. The program can be used in any center for spine surgery where effective interaction between polyvalent medical and nursing teams is maintained.

## Introduction

In the recent decades, there has been a remarkable increase in using minimally invasive technologies in spinal neuro-orthopedics [1, 2]. For patients with symptomatic degenerative diseases of the lumbar spine, the method of transforaminal interbody fusion with transpedicular stabilization is considered most appropriate [2]. On the one hand, minimally invasive dorsal interventions can reduce tissue damage and local pain during surgery; on the other hand, the long-term clinical and radiological outcomes of these techniques are not less successful than those of open decompression-stabilizing interventions [3, 4]. Considering the multilevel nature of vertebral pathology and the risks of polysegmental manipulations, it is crucial to further improve the treatment for degenerative diseases of the lumbar segments [5].

In current neurosurgery, a number of changes in the perioperative patient management are taking place [6, 7]. New approaches to analgesia in combination with stress-relieving techniques help reduce the number of complications and days of inpatient treatment [8, 9]. This strategy is based on the concepts of fast-track and ERAS (Enhanced Recovery After Surgery) [10]. In those, the patient management protocols incorporate the results of large-scale studies with high-class evidence, that are recommended for use by professional communities [11–13].

The implementation of such a multidisciplinary approach is made possible thanks to the continuous patient management at the prehospital stage, in the hospital setting, and during the outpatient postoperative follow-up [12]. This approach ensures a decrease in the severity of operation-associated stress, accelerates patient rehabilitation, and reduces the financial burden on practical healthcare [13, 14].

There is insufficient information on the use of accelerated recovery programs (ARPs) in spinal surgery. There are no specific ERAS recommendations for the treatment of patients with degenerative diseases of the spine.

Since 2017, Road Clinical Hospital (Irkutsk, Russia) has been using a multidisciplinary approach to accelerated rehabilitation of patients after spinal interventions, that is based on the continuous interaction between outpatient, inpatient, and rehabilitation procedures.

Evaluation of the effectiveness of this accelerated recovery program for patients with polysegmental degenerative diseases of the lumbar spine became **the aim of this study.**

## Materials and Methods

This was a longitudinal, prospective, single-center, non-randomized study. We analyzed the results of surgical treatment of 53 patients operated by using two-level transforaminal interbody fusion in segments L_II_–S_I_ from December 2017 to December 2019 at the Center for Neurosurgery and the Department of Anesthesiology and Resuscitation of Road Clinical Hospital (Irkutsk, Russia).

The study inclusion criteria were: the presence of lower back pain and radicular clinical symptoms due to degenerative disease of the lumbar spine, the involvement of two adjacent vertebral segments, and the absence of improvement after conservative treatment for 6–8 weeks.

The exclusion criteria included: single-level degenerative lesions of the lumbar spine; the ASA physical status degree above class III; a history of previous spinal surgery; the presence of concomitant diseases of the lumbar spine (infectious or inflammatory diseases, tumors, traumatic injuries), a significant decrease in bone mineral density (osteoporosis), any concomitant disease in the stage of decompensation, as well as intolerance to the medications used.

Patients were divided into 2 representative groups: in group 1 (n=24), dorsal decompression and stabilization surgeries were performed using the ARP; in group 2 (n=29), rigid stabilization from the posterior median approach with traditional intravenous anesthesia and artificial ventilation was used. The results of surgical treatment were followed-up: in group 1, for 18 [12; 22] months, in group 2, for 18 [14; 25] months. The study was conducted in accordance with the Declaration of Helsinki (2013) and approved by the Ethics Committee of the Irkutsk State Medical University (Russia).

All surgical interventions were performed by the operating team with more than 15 years of experience in open and minimally invasive dorsal interventions. All operated patients were under intensive supervision of one anesthesiologist. The program conceptualization was carried out by a group of specialists (spinal surgeons, anesthesiologist, neurologist, physiotherapist, and nurses) who were familiar with the basic principles of ARP.

In this clinical study, the accelerated recovery program included a close and continuous interaction between three stages of treatment: outpatient, inpatient, and rehabilitation (Table 1).

**Table 1:** Perioperative management of patients with polysegmental diseases of the lumbar spine

Criterion	ARP-guided dorsal decompression and stabilization	Traditional dorsal decompression and stabilization	Specialist in charge
***Outpatient-ambulatory stage***			
*Patient informed consent* Discussion about the surgery and anesthesia, possible risks and complications; presentation of similar clinical examples and demonstration of video footage of relevant operations — to ensure the patient’s understanding and psychological readiness for the upcoming surgery	Outpatient	In the hospital	Neurosurgeon, anesthesiologist
*Examination by an anesthesiologist* Examination of the clinical and instrumental data needed to identify the patient’s potential to compensate for the comorbid conditions and the need for correction	Outpatient	In the hospital	Anesthesiologist
*Quit smoking* Ruling out the effect of nicotine on the rheological properties of blood and protecting against smoking-induced intoxication	Several weeks before surgery	No	Anesthesiologist, neurosurgeon
*Hospitalization* Preoperative stay in the hospital	On the day or the eve of the operation	3–5 days before surgery	Neurosurgeon
***In-hospital stage (preoperative)***			
*Food and fluid intake* Discontinuation food and fluid intake to reduce the general stress of the patient’s body	Stop taking solid food 6 h before surgery, fluids — 2 h before surgery	Stop taking solid food 18 h before surgery, fluids — 10 h before surgery	Anesthesiologist
*Premedication* Reducing the drug burden on the patient and accelerating the rehabilitation process	Only in the presence of somatic disease	Midazolam, Promedol, Sibazon	Anesthesiologist
*Prevention of infectious complications* Use of antibacterial drugs	Antibiotic prophylaxis 2 h before the first incision	Antibiotic therapy	Anesthesiologist, neurosurgeon, clinical pharmacologist
*Prevention of thromboembolic complications* The use of compression hosiery. Ultrasound examination of lower limb veins before and the next day after surgery	Yes	Yes	Anesthesiologist, neurosurgeon, sonologist
***In-hospital stage (intraoperative)***			
*Anesthetic management:* multimodal analgesia using non-steroidal anti-inflammatory drugs prior to skin incision and before suturing — to reduce the need for analgesics; using dexmedetomidine to help control the depth of anesthesia, the restoration of consciousness, prevention of cognitive dysfunctions, and reduction of analgesics dosing; use of sugammadex for fast and effective reversal of the neuromuscular block upon patient extubation in the operating room	Yes	No (only traditional anesthesia with arduan, propofol, and fentanyl)	Anesthesiologist
*Surgical technique:* the use of minimally invasive surgical technologies (operating microscope, tubular retractor systems, transcutaneous surgical techniques, specialized micro-instruments, low-traumatic stabilizing systems) — to reduce iatrogenic damage to paravertebral tissues; infiltration of local anesthetics around the surgical wound before suturing to reduce the need for analgesics	Yes	No (open interventions with the median access)	Neurosurgeon
***In-hospital stage (postoperative)***			
*Use of drains* Prevention of infections and pain in the area of surgery	No drain or its early removal (on day 1)	Mandatory drain for 2–3 days	Neurosurgeon
*Use of a urinary catheter* Enabling early activation, reducing patient discomfort	Removing the urinary catheter in the operating room	After transferring to the post-surgery ward	Anesthesiologist
*Postoperative pain relief* Reducing the need for opioid analgesics and preventing their adverse effects	Multimodal approach	Common use of opiates	Anesthesiologist, neurosurgeon
***Rehabilitation stage (in hospital)***			
*Massage, physiotherapy* Accelerating the rehabilitation process	Upon first hours after surgery and recovery from post-anesthetic depression	After transferring to the post-surgery ward	Physiotherapist, massage therapist
*Verticalization* Prevention of thromboembolic and hypostatic complications	Within the first 12 h after surgery	On the 2^nd^ day after surgery (more than 24 h)	Neurosurgeon, physiotherapy specialist
*Physiotherapy* Using physiotherapeutic techniques to improve tissue microcirculation in the area of surgery, reducing postoperative edema to expand the range of motion	On the 1^st^ day after surgery	On the 2^nd^ day after surgery (more than 24 h)	Neurosurgeon, physiotherapist
*Sitting down* Improving patient comfort, accelerating rehabilitation	1–2 days after surgery	10–14 days after surgery	Neurosurgeon, physiotherapy specialist
***Rehabilitation stage (in a specialized rehabilitation hospital)***			
*Complex of rehabilitation measures* Walking, massage, and physiotherapy	Yes	Yes	Expert in rehabilitation, physiotherapist
***Outpatient-ambulatory stage***			
*Dynamic observation* Study of neurological and orthopedic status to determine the recovery of working capacity	Yes	Yes	Neurosurgeon, neurologist

We analyzed the number of bed-days spent after the operation, the number of perioperative surgical complications and adverse consequences of anesthesia, and the number of re-hospitalizations within 90 days. Additionally, we assessed the time of verticalization, the level of pain in the operated zone according to the visual analogue scale (VAS), the quality of life of patients in the long-term postoperative period (median 18 months) according to the SF-36 questionnaire (physical and psychological components of health).

**Statistical processing** of the results was performed using the Statistica 8.0 software. The normality of the data distribution was assessed using the Shapiro–Wilk, Kolmogorov–Smirnov, and Lilliefors tests. The distribution was considered deviating from normal if the above tests indicated statistically significant differences (p<0.05). For non-normal distributions, nonparametric statistics were used to assess the significance. Differences between the groups were considered statistically significant at p<0.05. The results are presented by the median and the values of the 1^st^ and 3^rd^ quartiles — Me [25; 75]. To compare and analyze the data, we used the Mann–Whitney U-test and the Wilcoxon test for nonparametric data and the χ^2^ test for binomial signs.

## Results

Patient characteristics are presented in Table 2. There were no significant differences between values of the parameters studied (p>0.05).

**Table 2:** Patient characteristics

Indicators	Group 1 (n=24)	Group 2 (n=29)
Age (years) (Me [25; 75])	58 [44; 69]	55 [41; 68]
Gender:		
female/male	10/14	11/18
р±σ_р_% male	58.3±8.9	62.1±11.3
Body mass index (Me [25; 75])	25.9 [23.2; 27.3]	24.5 [23.6; 26.8]
Concomitant disease, n (%):		
diabetes	1 (4.15)	2 (6.9)
arterial hypertension	2 (8.3)	3 (10.35)
kidney diseases	1 (4.15)	1 (3.45)
lung diseases	1 (4.15)	1 (3.45)
coronary artery disease	2 (8.3)	2 (6.9)
Localization of operated segments, n (%):		
L_II_–L_III_–L_IV_	1 (4.15)	2 (6.9)
L_III_–L_IV_–L_V_	6 (25.0)	7 (24.15)
L_IV_–L_V_–L_VI_	4 (16.7)	5 (17.2)
L_V_–L_VI_–S_I_	2 (8.3)	1 (3.45)
L_IV_–L_V_–S_I_	11 (45.85)	14 (48.3)

Comparative analysis of the results showed that in the ARP group, there were significantly (p<0.05) lower values of such parameters as the duration of surgery and anesthesia, the volume of blood loss, the amount of injected opioids, the time of verticalization, and the duration of inpatient treatment (Table 3). Notably, the inpatient treatment in the ARP group included early rehabilitation measures provided directly in the specialized clinic by a physiotherapy specialist and a massage therapist.

**Table 3:** Data on surgical operation and postoperative management (Me [25; 75])

Indicators	Group 1 (n=24)	Group 2 (n=29)
Duration of operation (min)	168 [126; 195]*	256 [208; 324]
Duration of anesthesia (min)	185 [130; 210]*	270 [215; 340]
Blood loss volume (ml)	75 [50; 130]*	180 [70; 260]
The number of agents administered for anesthesia, 0.005% fentanyl (ml/case)	20.0 [12.0; 23.5]*	31 [20.5; 32.5]
Verticalization time (days)	1 [1; 2]*	2 [1; 2]
Duration of inpatient treatment (days)	9 [7; 9]*	10 [10; 12]

* p<0.05 between the groups at the same stages of the study.

**Figure 1:**
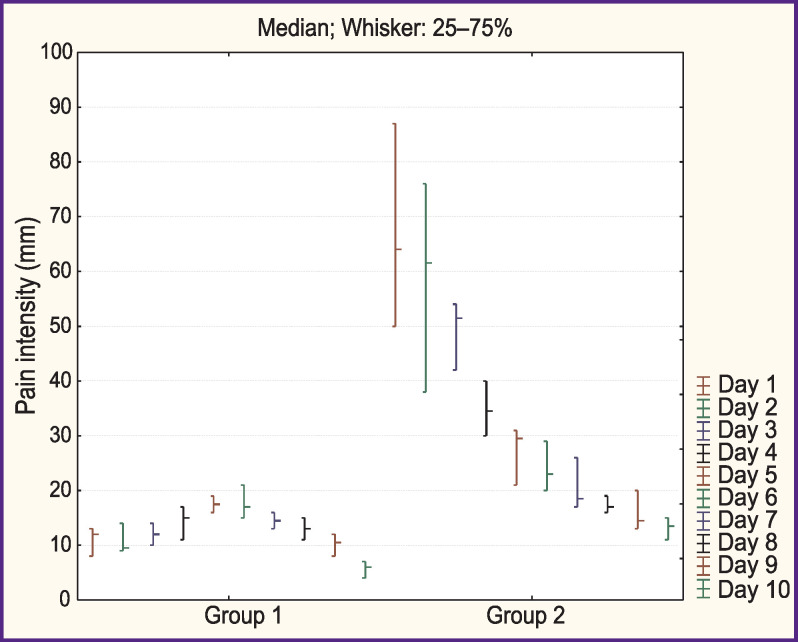
Severity of pain in the area of surgery as assessed with the visual analogue scale

Pain assessment according to the VAS showed a significantly lower (p<0.05) level of pain in the operated zone among patients under ARP during the entire period of inpatient treatment (Figure 1).

**Figure 2:**
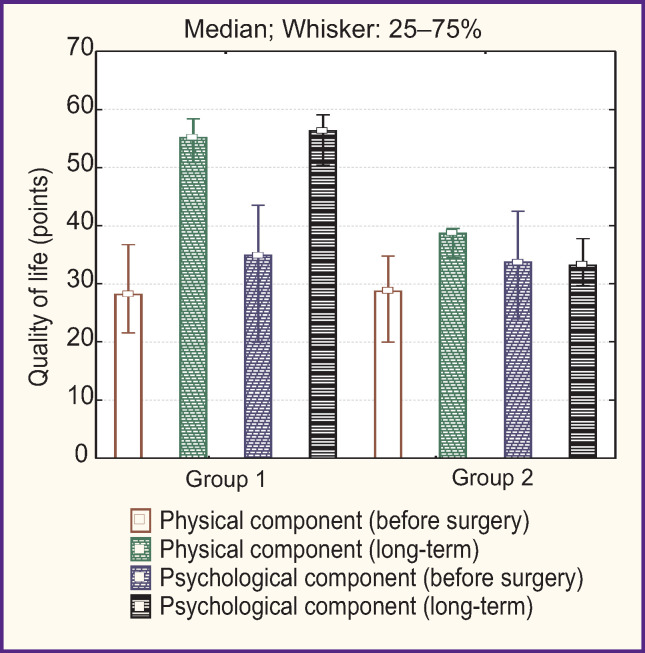
Quality of life according to the SF-36 questionnaire

When studying the quality of life of patients using the SF-36 questionnaire, a statistically significant improvement in the physical and psychological components of health was found in both groups (Figure 2). In the long-term postoperative period, the quality of life indicators was significantly better in patients treated according to the ARP (p<0.05).

Patients under ARP had no adverse effects of anesthesia that made their stay in the intensive care unit any longer (p<0.05) (Table 4). In the control group though, 5 patients (17.2%) experienced slow restoration of neuromuscular conduction, which required a longer than usual stay in the intensive care unit.

**Table 4:** Adverse effects of anesthesia, n (%)

Adverse effect	Group 1 (n=24)	Group 2 (n=29)
Vomiting	—	1 (3.45)
Bradycardia	1 (4.15)	—
Respiratory depression	—	1 (3.45)
Dizziness	1 (4.15)	2 (6.9)
Nausea	1 (4.15)	2 (6.9)
Extra time to recover from neuromuscular block	—	5 (17.2)

A greater number of verified postoperative complications was noted in group 2 as compared to group 1 (p=0.003) (Table 5). During the 90-day postoperative observation, 4 patients of group 2 (13.8%) needed to be referred to a medical institution for additional care. In patients of group 1, there were no complications that required re-hospitalization.

**Table 5:** Surgical postoperative complications, n (%)

Complication	Group 1 (n=24)	Group 2 (n=29)
Surgery site infection	—	2 (6.9)
Intermuscular hematoma	—	1 (3.45)
Damage to the dura mater	—	—
Venous thromboembolic complications	1 (4.15)	1 (3.45)
Adjacent segment disorder	—	1 (3.45)
Pseudoarthrosis	1 (4.15)	—
Instability of the fixing device	—	1 (3.45)

## Discussion

Numerous studies have confirmed the effectiveness of the ERAS protocol in various areas of surgery [11, 12]. However, there are currently no specific instructions for the implementation of individual elements of the ERAS protocol in spinal surgery. Smith et al. [15] believe that when performing one- or two-level lumbar fixation, multimodal anesthesia (including preoperative administration of acetaminophen and gabapentin) is indicated. In addition, they recommend using early postoperative verticalization and physiotherapy, as well as prevention of postoperative nausea and vomiting. In this study, however, the authors did not comment on the need for intraoperative management of water-electrolyte balance and hemodynamics.

According to Ren et al. [16], the ERAS program for posterior lumbar fusion should include preoperative patient updating, preoperative bowel preparation, and preoperative fasting, optional fluid intake, intraoperative body temperature monitoring, the use of short-acting anesthetic drugs and preoperative antibiotic therapy, control of passage through the digestive tract to prevent nausea and vomiting, as well as early extubation.

Soffin et al. [17] recommend the following approach to ERAS for minimally invasive lumbar decompression:

before surgery — preventive analgesia, pre-surgery discussion with the patient, the minimum period of preoperative fasting, prevention of nausea and vomiting;

during surgery — the standard anesthetic protocol using ketamine or propofol, opioid-sparing multimodal anesthesia using ketorolac or lidocaine, minimally invasive surgical technique, no drainage of the wound and catheterization of the bladder, maintenance of normothermia and normovolemia, antibiotic prophylaxis, prevention of nausea and vomiting;

after surgery — early nutrition, early verticalization, opioid-sparing analgesia.

Licina et al. [9] suggest 22 ARP criteria that can potentially be used in spinal surgery:

before hospitalization — preoperative discussion with the patient, risk stratification and modification of lifestyle factors (alcohol, smoking), strengthening of the musculoligamentous system, preoperative nutrition, preoperative control of anemia, preoperative carbohydrate load, preventive analgesia;

in the intraoperative period — perioperative blood preservation procedures, minimally invasive surgical approaches, antibiotic prophylaxis, local infiltration anesthesia, use of the anesthesia protocol, prevention of nausea and vomiting, maintenance of normothermia, maintenance of water balance, preventing perioperative analgesia;

in the postoperative period — prevention of thrombosis, abandoning bladder catheterization, postoperative nutrition and maintenance of water balance, glycemic control, early activation, audit.

However, the authors emphasize the need for additional confirmation of the effectiveness of these criteria based on the results of randomized clinical trials and meta-analyses.

Along with the active implementation of lumbar spine decompression and stabilization techniques, there are significant variations reported about perioperative complications, duration of hospitalization, intensity of postoperative pain, and the functional outcome. These variations may be due to different surgical approaches and anatomical corridors, the use of different stabilizing structures, and a varying experience of the surgical team [18, 19]. In this regard, the unification of multidisciplinary approaches to surgical treatment and rehabilitation is needed to increase the clinical and economic effectiveness of specialized medical care. Reducing financial costs is achieved mainly by reducing unnecessary perioperative procedures, preventing complications, and improving patient management [20].

In this study, we used the standard criteria of the commonly accepted ERAS protocol for general surgery with beneficial effects confirmed by systematic reviews and meta-analyses, as well as by large cohort studies. Here, these criteria were applied to two-level decompression-stabilizing dorsal interventions in patients with polysegmental degenerative diseases of the lumbar spine. The study also focused on the continuity of outpatient, inpatient and rehabilitation procedures to achieve the fastest social and vocational rehabilitation of patients.

Effective management of perioperative analgesia is an important factor of the comprehensive ERAS program aimed at accelerating the recovery of patients after spinal surgeries. Thus, according to the data of Wang et al. [7], the use of sedation and infiltration anesthesia can improve the functional performance and reduce the number of complications during decompression and stabilization interventions. Soffin et al. [17] emphasize the priority of general anesthesia during minimally invasive lumbar decompression to ensure adequate pulmonary ventilation, restriction of patient’s movement, and an optimal degree of analgesia.

The minimally invasive surgical technology allows one not only to improve clinical indicators but also to reduce the duration of inpatient treatment. Chang et al. [4] applied the ERAS protocol of minimally invasive transforaminal fusion with endoscopic assistance and intravenous sedation to one- and two-level decompression-stabilizing interventions. This approach made it possible to significantly reduce the need for opioid analgesics; the duration of inpatient treatment was also reduced by about 1.4 days compared to conventional rigid stabilization [4]. Nazarenko et al. [21] found that the use of ARP for microdiscectomy reduced the severity of pain in the early postoperative period by 10% and improved the functional recovery by 20% compared to the standard perioperative management of patients with herniated intervertebral discs.

In addition to reducing the intensity of postoperative pain syndrome and shortening the duration of hospitalization, the purpose of using the ERAS protocols is to prevent the development of adverse effects of opiates, such as respiratory failure, bowel dysfunction, nausea, vomiting, and urinary retention [22]. The use of minimally invasive surgical technologies for dorsal rigid stabilization can reduce the period of inpatient treatment in comparison with the open decompression-stabilization techniques (3.4 and 5.1 days, respectively; p<0.02) and reduce the need for opioid analgesics (37.5 and 49.5 mg morphine per day, respectively; p<0.015) [23]. Cheng et al. [24] compared patients who underwent single-level transforaminal fusion by minimally invasive vs open intervention; the minimally invasive technique was found superior in terms of the need for opiates — 66.5 vs 201.5 mg/day (p=0.019), the hospitalization period — 4.80 vs 6.05 days (p=0.006), and the financial efficiency.

Today, the ERAS concept is the subject of active discussions in the professional community. Most specialists advocate the introduction of less invasive surgical technologies, outpatient surgical care, and opioid-sparing anesthetics as means for increasing the efficiency of practical healthcare [25, 26].

Corniola et al. [27] conducted an online survey of 234 members of the EANS (European Association of Neurosurgical Societies), including 9 questions about ERAS protocols in spine surgery. Regarding the ERAS protocols for spine surgery, 54.7% of the survey participants had no idea about ARP in spinal surgery, but the other 36% of the respondents confirmed that they did actively use ERAS elements in their practice. Therefore, in order to popularize the rapid recovery protocol in vertebrology, it is necessary to ensure that specialists are aware of the effectiveness of ARP in patients with spinal diseases.

In our study, it was found that the use of the ARP program during two-level dorsal decompression and stabilization allowed us not only to improve the clinical outcomes, but also to reduce the number of readmissions, the incidence of surgical perioperative complications, and adverse drug effects.

A significant limitation of the present study is its single-center setting and small numbers of patients. Also, we did not consider a possible influence of various stabilizing devices on the parameters studied in the intra- and perioperative periods.

## Conclusion

In this study, the accelerated recovery program has shown its safety and high clinical efficacy for patients with degenerative diseases of the lumbar spine.

The proposed program can be used in any center for spinal surgery to boost the multidisciplinary efforts of neurosurgeons, anesthesiologists, neurologists, clinical pharmacologists, physiotherapists, and specially trained nursing staff.

The active introduction of personalized medicine and the development of protocols for high standard medical care for patients undergoing spine surgery will improve the quality and reduce the cost of specialized medical services.
